# Renal infarction associated with low dose intravenous immunoglobulin in a kidney transplant recipient with sepsis: a case report and literature review

**DOI:** 10.1186/s12882-021-02545-1

**Published:** 2021-10-13

**Authors:** Eun Woo Choi, Jun Young Do, A. Young Kim, Seok Hui Kang

**Affiliations:** 1 Division of Nephrology, Department of Internal Medicine, Dongkuk University Medical Center, Gyeongju, Republic of Korea; 2 Division of Nephrology, Department of Internal Medicine, College of Medicine, Yeungnam University, Daegu, Republic of Korea; 3 Department of Internal Medicine, Yeungnam University Medical Center, 317-1 Daemyung-Dong, Nam-Ku, Daegu, 705-717 South Korea

**Keywords:** Renal infarction, Intravenous immunoglobulin, Thromboembolism

## Abstract

**Background:**

The use of human intravenous immunoglobulin gamma (IVIG) is associated with thromboembolic events as a complication. There are few reported cases of renal infarction during IVIG use in the general population, but transplant kidney may be more susceptible to thromboembolic events following IVIG use.

**Case presentation:**

A 41-year-old woman visited with fever and pain at the transplant kidney. Six years ago, she underwent kidney transplantation from a deceased donor. Laboratory and radiologic findings were compatible to septic condition, secondary to acute pyelonephritis. We started antibiotics, inotropics, and IVIG. The patient abruptly developed gross hematuria and urine output decreased to 100 cc/day during IVIG administration. Renal doppler and pathologic findings revealed renal infarction. Oliguria and azotemia persisted and she is undergoing maintenance hemodialysis.

**Conclusion:**

Our case shows that infarction of transplant kidney can be caused by IVIG use in a patient with severe infection. Thus, when using IVIG for kidney transplant patients with high risk of thromboembolic events, we may be careful to prevent the thromboembolic events.

## Background

Human intravenous immunoglobulin gamma (IVIG) is used to immunological and anti-inflammatory treatment. IVIG has been used in primary and secondary immunodeficiency disorders, neuromuscular, infectious, autoimmune, and inflammatory disease treatment. In recent decades, the use of IVIG has been extended to the role of organ transplantation [1, 2]. IVIG therapy is generally considered safe, but the reported side effect rate of IVIG is 10–30% [3]. Adverse effects include anaphylactic/anaphylactoid reactions, renal and pulmonary complications, thrombosis/embolism, colitis, and bloodborne infectious disease [4–6]. Thromboembolic events, a late and severe side effect of IVIG, including myocardial infarction, pulmonary embolism, cerebrovascular accident, deep vein thrombosis and hepatic veno-occlusive disease, are life-threatening [4]. Other thrombotic adverse events have been reported in the use of IVIG. However, renal infarction due to IVIG use has not been reported and grafted kidney infarction following use of IVIG has only been reported in one case, following desensitization in kidney transplantation [7]. However, there are no reports about graft infarction after use of low-dose IVIG. In this report, we present a case of grafted kidney infarction after IVIG infusion in a kidney transplant patient with severe sepsis.

## Case presentation

A 41-year-old woman visited with fever and pain in the transplant kidney. She had end-stage renal disease due to unknown cause and underwent peritoneal dialysis for 7 years. The patient was diagnosed with azotemia 14 years ago. During her first visit, both her kidneys presented with decreased size (approximately 7.8 cm) and increased echogenecity, and her serum creatinine level was 397.8 μmol/L. Considering these, we diagnosed her with end-stage renal disease and did not perform renal biopsy to confirm any underlying disease. Before dialysis, we performed autoimmune studies, checking for anti-nuclear antibodies, anti-double-strand DNA antibodies, C3, C4, anti-glomerular basement membrane antibodies, or anti-neutrophil cytoplasmic autoantibodies, but all of them showed no evidence of autoimmune disease. In addition, the patient did not present any symptoms or signs associated with coagulopathies; her bleeding time was 1.3 min (reference range: 1–4 min). Six years ago, she underwent kidney transplantation from a deceased donor. After kidney transplantation, she received 5 mg prednisolone, 4 mg tacrolimus, and 1500 mg mycophenolate mofetil as immunosuppressants and 2.5 mg amlodipine for hypertension. Her tacrolimus trough level remained stable between 6.2 and 8.7 nmol/L. There was no rejection history. Her serum creatine level was 71.6 μmol/L, and renal graft function was normal on outpatient examination.

**Fig. 1:**
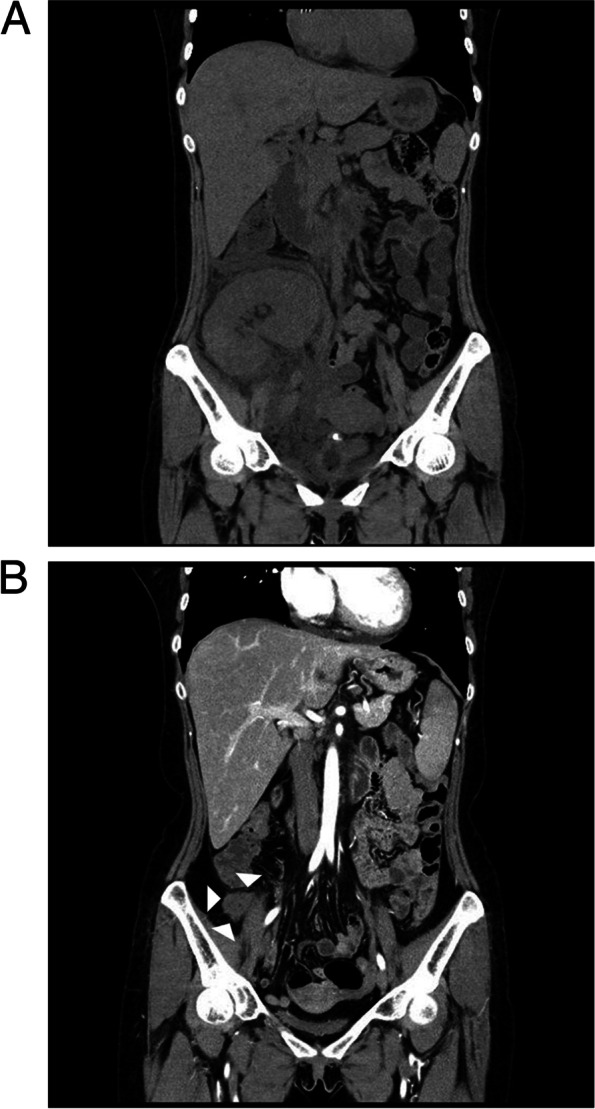
Changes in transplant kidney according to clinical courses. **A** At onset of acute pyelonephritis, peri-renal infiltration and fluid collection around transplant kidney in non-contrast image revealed acute pyelonephritis. **B** After 4 years of renal infarction, contrast image showed markedly atrophied transplant kidney (arrow head)

At 6 years after the transplantation, she visited the hospital, complaining of fever and pain in the grafted kidney. Her blood pressure was 80/50 mmHg, and her body temperature was 39.5 °C. She had direct and rebound tenderness over the grafted kidney. The urine output on the first hospital day was only 100 cc/day. She was admitted to intensive care, and her electrocardiogram showed tachycardia with normal sinus rhythm. On admission, laboratory analysis showed a white blood cell (WBC) count of 1381 × 10^9^/L, hemoglobin level of 134 g/L, platelet count of 90 × 10^9^/L, and C-reactive protein level of 32.0 mg/dL. Her blood urea nitrogen and serum creatinine levels were 10.7 mmol/L and 435.8 μmol/L, respectively. The lactate dehydrogenase level was 607 IU/L (reference range: 150–550 IU/L). Urine microscopy showed 5–10 WBCs/high power field (HPF) and 3–5 red blood cells/HPF. Her tacrolimus level on admission was 6.2 nmol/L. BK virus were determined on admission and the virus titer then was < 100 copies/mL in her urine or whole blood. Her thyroid-stimulating hormone and free T4 levels were 1.66 mIU/L (reference range: 0.34–4.25 mIU/L) and 10.35 pmol/L (reference range: 10.3–21.9 pmol/L), respectively. Her basal adrenocorticotropin and cortisol levels were 3.5 pmol/L (reference range: < 26 pmol/L) and 423.7 nmol/L (reference range: 138–690 nmol/L), respectively. Non-enhanced computed tomography was performed immediately after admission and revealed acute pyelonephritis (APN) (Fig. 1A). In addition, our center performed a multiplex PCR for her using a nasal swab, which is performed for detecting respiratory virus in patients with a septic condition; the PCR kit detects respiratory viruses, including influenza virus, respiratory syncytial virus, human metapneumovirus, adenovirus, human coronavirus, human enterovirus, human bocavirus, parainfluenza virus, and human rhinovirus [8]. All viruses were not detected in our case.

Based on clinical and laboratory tests, we used ceftriaxone 2 g per day and inotropics for septic shock and APN. On the first hospital day (HD1), she had oliguria (100 cc/day), and we started emergency hemodialysis. We treated her with IVIG (Liv Gamma; SK Chemical Life Science, South Korea) for septic shock (300 mg/kg/day for 3 days). On HD2, her blood pressure stabilized with use of inotropic agents and fluid therapy, and urine output increased to 400 cc/day. However, on the second day (HD3) of IVIG administration, she abruptly developed gross hematuria and urine output decreased to 100 cc/day. At that time, we performed additional laboratory tests to differentiate the other causes of hematuria. Her platelet counts were normal at 174 × 10^9^/L, and prothrombin time and activated partial thromboplastin time also showed normal values of 10.4 s and 34.1 s, respectively. On peripheral blood smear, microangiopathic hemolytic anemia finding was not seen. The patient underwent continuous electrocardiogram monitoring during IVIG infusion; however, there was no evidence of arrythmia. In addition, on HD 3, further heart problem was evaluated using electrocardiogram and echocardiogram. The electrocardiogram showed normal sinus rhythm without ST-T changes, whereas the echocardiogram revealed left ventricular ejection fraction decreased to 34%, and a borderline enlarged left ventricle. Echocardiogram also showed abnormalities in the basal to upper middle left ventricular regional wall motion, which is more likely to cause stress induced cardiomyopathy than acute coronary syndrome. Her abnormal echocardiographic findings were considered transient, caused by stress induced cardiomyopathy owing to septic shock.

**Fig. 2:**
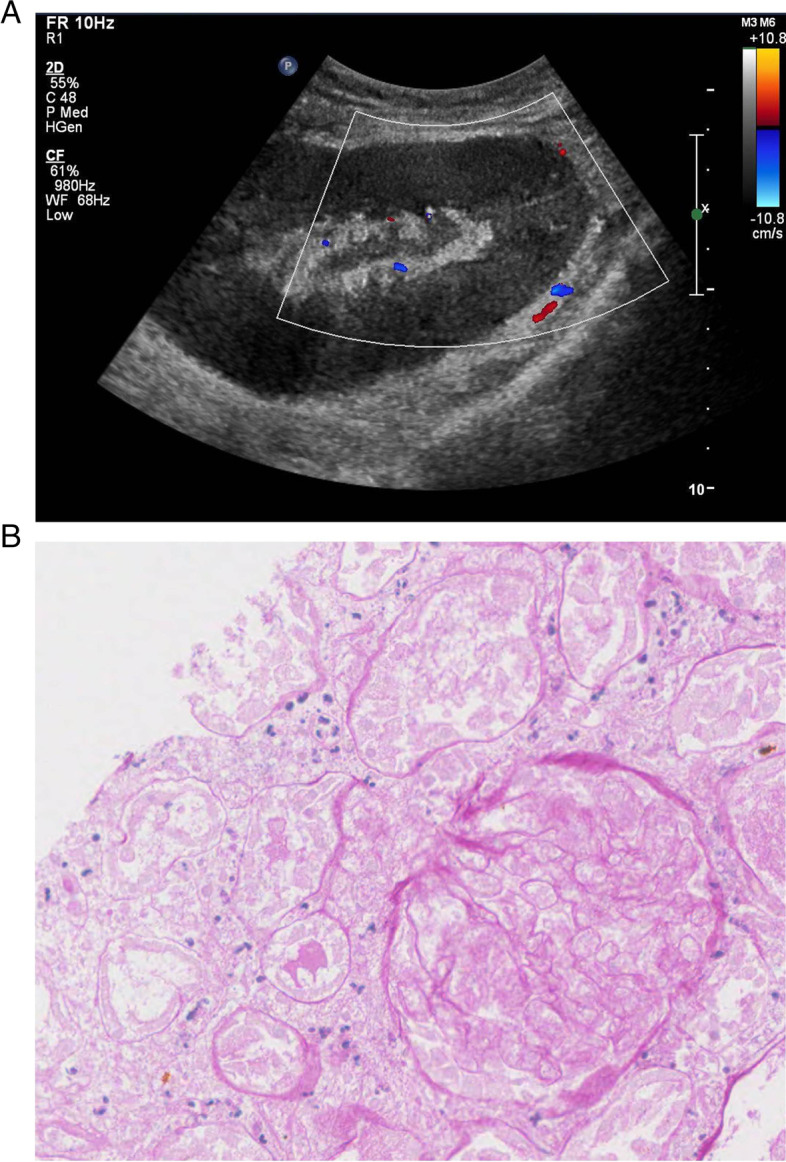
Renal doppler and pathologic findings. **A** Flow signal at renal parenchyma was not detected in renal doppler. **B** Periodic acid-Schiff stain of the kidney showed coagulative necrosis with glomerular and tubular cells without discernible nuclei (× 400)

IVIG treatment was terminated at HD4, but oliguria and gross hematuria persisted. We considered a kidney biopsy to evaluate oliguria and hematuria, but could not proceed because her serious condition. The fever was persisted so the antibiotic was changed to meropenem. On HD 4, vancomycin added due to persistent fever following antibiotic change. On HD7, the fever disappeared, but oliguria persisted. LDH levels on HD 9 and 14 increased to 1704 and 1258 IU/L, respectively, compared to 607 IU/L at the time of admission. Blood and urine cultures were repeatedly performed since she was admitted, but no growths were identified in the two specimens. Her first transfusion was performed due to her low hemoglobin level of 76 g/L at HD 22. On HD 14, we performed conventional kidney sonography, which showed thickening of the renal pelvis and urethral wall; it did not show abnormal focal lesions and hydronephrosis. However, on HD 29, we performed doppler ultrasonography to confirm renal infarction and observed a lack of renal parenchymal and hilar vascular flow (Fig. 2A). We also performed renal biopsy and reveal that renal infarction with diffuse ischemic changes in the glomeruli but no rejection or other pathology (Fig. 2B). In addition, we performed SV40 staining using the renal biopsy specimen, and the staining results were negative for SV40. The biopsy specimen did not include medium or large vessels, and arteriolopathies, such as hyalinosis in small arteries or arterioles, were not detected within specimens. After discharge, oliguria and azotemia persisted and she is still on hemodialysis. Follow-up echocardiogram after discharge showed normal wall motion and improved left ventricular ejection fraction (71%). Her bleeding time and autoimmune studies were performed again 2 months after discharge. Her bleeding time was 2 min, and autoimmune studies once again showed negative findings. These results also did not show any evidence of coagulopathies or any autoimmune diseases as underlying comorbidities. Contrast enhanced computed tomography showed markedly atrophied transplant kidney after 4 years of renal infarction (Fig. 1B).

## Discussion and conclusion

This case report describes grafted kidney infarction due to IVIG administration for the treatment of APN and severe sepsis. We used IVIG to increase serum bactericidal action and for modulation of cytokine release and immunomodulatory effects [9]. There are few reported cases of renal infarction during IVIG use in the general population. Grafted kidney infarction following APN has only been reported in one case in a patient with atrial fibrillation [10]. There is only one reported case of hemorrhagic infarction with graft rupture due to high-dose IVIG use for desensitization in kidney transplantation. However, grafted kidney infarction after low dose IVIG following APN with sepsis has not been reported yet.

A review of thrombotic adverse events related to the administration of IVIG between 2008 and 2010 showed that 1% of patients developed these side effects [11]. Thrombotic events are triggered by an increase in plasma viscosity, activation of procoagulant factors or coagulation factors in the IVIG not removed by fractionation, vasospasm, autoimmune vasculitis, and an increase in the platelet count or adhesiveness. Among these, an increase in viscosity is the largest contributor to the occurrence of thrombotic events [12, 13]. Increasing plasma viscosity is associated with IgG in IVIG [14]. Risk factors for thrombotic adverse events due to IVIG administration include a large first infusion, oral contraceptive use, prior/current thrombosis, preexisting atherosclerotic disease, elevated serum viscosity, a hereditary hypercoagulable state or idiopathic thrombocytopenic purpura, age >  45 years old, prior thrombotic events, and a hypercoagulable state, such as infection [11, 15, 16].

Renal infarction during IVIG use has not been reported yet in the general population. However, one case of rupture with hemorrhagic infarction in a grafted kidney was reported [7]. The current case also occurred in the presence of grafted kidney infarction during IVIG administration. Although not reported in the general population, two such cases have been reported in grafted kidneys, suggesting that the grafted kidney is more susceptible to thromboembolic events. As seen in this case, thrombotic events can even occur during low-dose IVIG use in the presence of multiple risk factors and a grafted kidney (Table 1) [17, 18]. It is important to note that kidney transplantation per se may already be prone to formation of thrombosis due to artificial anastomosis of the recipient and donor vessels. This vulnerability is thought to be associated with turbulent blood flow in the grafted kidney. Furthermore, in our case, other factors may have been associated with renal infarction during IVIG administration. Septic shock is a well-known hypercoagulable state induced by the activation of the coagulation system [19]. In addition, sepsis with severe inflammation is associated with changes in the endothelial cells, which produce an anticoagulation or profibrinolytic effect, volume depletion, and hypotension. Hypertension as an underlying comoridity, and immobilization could be associated with the stasis of blood flow and endothelial injuries. In relation to these, the use of calcineurin inhibitors or vasoactive drugs is also associated with vasoconstriction and platelet aggregation [20].

**Table 1:** Risk factors of and interventions for preventing allograft infarction during IVIG administration in kidney transplant recipients

Risk factors	Preventive interventions during IVIG	Diagnosis
Old age (> 45 years old)	Hydration before and after administration	Dopplex ultrasound
History of prior thrombotic events	Slow infusion	Contrast enhanced CT
Immobilization	Limitation of daily dose of IVIG (< 400-500 mg/kg)	Radioisotope scan
Allograft causes	Use of aspirin or LMWH (considering risk vs. benefit)	Angiography
Arterial kinking or torsion		
End-to-end anastomosis of artery		
Multiple renal arteries of allograft		
Trauma		
Hypercoagulability		
Infection (esp, sepsis)		
Hypotension		
Hemolytic uremic syndrome		
Drugs (e.g., cyclosporine, oral contraceptives)		
Antiphospholipid syndrome		
Genetic mutations (e.g., factor V Leiden)		
Comorbidities		
Cardiac problem (e.g., atrial fibrillation)		
Atherosclerosis		
Renal artery stenosis		
Diabetes mellitus		
Hypertension		
Vasculitis associated with endothelial damage		
Nephrotic syndrome		
Increased intra-renal pressure		
Acute tubular necrosis		
Hydronephrosis		
Acute rejection		

*Abbreviations*: *IVIG* intravenous immunoglobulin, *LMWH* low-molecular-weight heparin, *CT* computed tomography

In our case, renal infarction was confirmed on HD 29 by renal Doppler ultrasonography. However, clinical findings indicate that renal infarction may have developed on HD 3. First, the patient had straw-colored or clear urine but abruptly developed gross hematuria during IVIG infusion on HD3. Gross hematuria continued until HD 8 and was straw-colored or brownish urine was seen from HD 9 until discharge. Second, the urine output recovered to 400 cc/day on HD 2; however, it decreased to < 100 cc/day from the onset of gross hematuria. The urine output did not recover after the onset of gross hematuria, despite improvement in the general condition and laboratory findings of the patient. Although definite diagnosis did not coincide with the development of symptoms/signs, these clinical findings provide the evidence for development of renal infarction on HD 3. We did not suspect renal infarction prior to doing a renal doppler ultrasound. There were no specific changes on the infarcted kidney, and we did not perform contrast CT due to acute kidney injury. We performed conventional kidney sonography at HD 14, which showed thickening of her renal pelvis and urethral wall. However, it did not show any abnormal focal lesions and hydronephrosis. This reveals that in patients with sudden onset of gross hematuria, acute kidney injury, and laboratory findings indicating tissue destruction, physicians should to suspect the occurrence of renal infarction. For these patients, they should perform doppler ultrasound rather than conventional ultrasound.

The patient survived from severe infection, but her grafted kidney was failure. Prevention is the most important because the occurrence of these side effects causes irreversible complications. To reduce the risk of thrombosis when using IVIG, it is essential to identify the risk factors. The daily use of IVIG should be limited to 400–500 mg/kg, and hydration should be considered before and after administration. The use of premedications, such as aspirin or low-molecular-weight heparin, may be considered in high-risk patients if it is not contraindicated to use them. Slow infusion is also effective in preventing thrombosis. The use of a protocol that includes hydration, premedication, and slow infusion in renal transplantation patients reduces the risk of thrombosis [21]. Furthermore, in kidney transplant recipients prone to developing thromboembolic events, proper imaging surveillance should be considered to diagnose renal infarction during suitable times, especially if laboratory or clinical findings show any suspicious findings associated with renal infarction. Doppler ultrasonography or contrast CT should also be performed in patients with high risk of thromboembolic events rather than conventional ultrasonography or non-contrast CT due to their limitations in detecting renal blood flow.

There were inconsistent results regarding the association determined between the use of IVIG and clinical outcomes in septic patients. Although the meta-analysis using high quality trials alone did not show a significant effect on survival, the meta-analysis using all randomized trials showed a reduction in mortality [22, 23]. A recent trial has shown that high-dose IVIG (1.5-2.0 g/kg) is associated with favorable outcomes [24]. Considering these, the current guidelines state that the use of IVIG in septic patients is supported by weak recommendations or weak evidence [25, 26]. However, the use of IVIG can be considered in septic patients who are also immunodeficient. In our study, the patient received three immunosuppressants and could not withdraw any of her medications. Although these medications preferentially work on T-cells, previous studies have shown that they can directly or indirectly attenuate humoral immunity [27–29]. There were few data regarding the efficacy of IVIG in septic patients who were kidney transplant recipients; the patient would have an acquired immunodeficient status compared to the general population. Our case shows that IVIG can be considered as a possible option of treatment in septic patients.

In conclusion, infarction of transplant kidney can be caused by IVIG use in a patient with sepsis, even with low-dose administration. Transplant kidney may be more susceptible to thromboembolic events following IVIG use. Thus, when using IVIG for kidney transplant patients with high risk of thromboembolic events, we may be careful to prevent the thromboembolic events.

## Data Availability

All data generated or analysed during this study are included in this published article.

## Abbreviations

IVIG: Human intravenous immunoglobulin gamma
APN: Acute pyelonephritis
HD: Hospital day

## References

[1] Shehata N, Palda VA, Meyer RM, Blydt-Hansen TD, Campbell P, Cardella C (2010). The use of immunoglobulin therapy for patients undergoing solid organ transplantation: an evidence-based practice guideline. Transfus Med Rev.

[2] Jordan SC, Tyan D, Stablein D, McIntosh M, Rose S, Vo A (2004). Evaluation of intravenous immunoglobulin as an agent to lower allosensitization and improve transplantation in highly sensitized adult patients with end-stage renal disease: report of the NIH IG02 trial. J Am Soc Nephrol.

[3] Sherer Y, Levy Y, Langevitz P, Rauova L, Fabrizzi F, Shoenfeld Y (2001). Adverse effects of intravenous immunoglobulin therapy in 56 patients with autoimmune diseases. Pharmacology.

[4] Stiehm ER (2013). Adverse effects of human immunoglobulin therapy. Transfus Med Rev.

[5] Orbach H, Katz U, Sherer Y, Shoenfeld Y (2005). Intravenous immunoglobulin: adverse effects and safe administration. Clin Rev Allergy Immunol.

[6] Brennan VM, Salome-Bentley NJ, Chapel HM (2003). Prospective audit of adverse reactions occurring in 459 primary antibody-deficient patients receiving intravenous immunoglobulin. Clin Exp Immunol.

[7] Sin YH, Kim YJ, Oh JS, Lee JH, Kim SM, Kim JK (2014). Graft rupture after high-dose intravenous immunoglobulin therapy in a renal transplant patient. Nephrology (Carlton).

[8] Lee EK, Lee YY, Choi KH (2013). Epidemiology and clinical features of respiratory viruses in pediatric inpatients in a single medical Center in Daegu from 2010 to 2012. Yeungnam Univ J Med.

[9] Shankar-Hari M, Spencer J, Sewell WA, Rowan KM, Singer M (2012). Bench-to-bedside review: Immunoglobulin therapy for sepsis - biological plausibility from a critical care perspective. Crit Care.

[10] Tsai SF (2014). The first case of atrial fibrillation-related graft kidney infarction following acute pyelonephritis. Intern Med.

[11] Daniel GW, Menis M, Sridhar G, Scott D, Wallace AE, Ovanesov MV (2012). Immune globulins and thrombotic adverse events as recorded in a large administrative database in 2008 through 2010. Transfusion.

[12] Bentley P, Rosso M, Sadnicka A, Israeli-Korn S, Laffan M, Sharma P (2012). Intravenous immunoglobulin increases plasma viscosity without parallel rise in blood pressure. J Clin Pharm Ther.

[13] Wolberg AS, Kon RH, Monroe DM, Hoffman M (2000). Coagulation factor XI is a contaminant in intravenous immunoglobulin preparations. Am J Hematol.

[14] Baba R (2008). Effect of immunoglobulin therapy on blood viscosity and potential concerns of thromboembolism, especially in patients with acute Kawasaki disease. Recent Pat Cardiovasc Drug Discov.

[15] Levi M, Schultz M, van der Poll T (2013). Sepsis and thrombosis. Semin Thromb Hemost.

[16] Paran D, Herishanu Y, Elkayam O, Shopin L, Ben-Ami R (2005). Venous and arterial thrombosis following administration of intravenous immunoglobulins. Blood Coagul Fibrinolysis.

[17] Allen RDM, Morris PJ, Knechtle SJ (2014). Vascular and lymphatic complications after kidney transplantation. Kidney transplantation –principles and practice.

[18] O’Bell JW, Bayliss GP, Dworkin LD, Coffman TM, Falk RJ, Molitoris BA, Neilson EG, Schrier RW (2013). Renal artery thrombosis, thromboembolism, atheroemboli, and renal vein thrmobosis. Schrier’s diseases of the kidney.

[19] Libby P, Simon DI (2001). Inflammation and thrombosis: the clot thickens. Circulation.

[20] Birk AV, Leno E, Robertson HD, Bolotina VM, Szeto HH (2003). Interaction between ATP and catecholamines in stimulation of platelet aggregation. Am J Physiol Heart Circ Physiol.

[21] Huang L, Kanellis J, Mulley W (2011). Slow and steady. Reducing thrombotic events in renal transplant recipients treated with IVIg for antibody-mediated rejection. Nephrology.

[22] Soares MO, Welton NJ, Harrison DA, Peura P, Shankar- Hari M, Harvey SE (2012). An evaluation of the feasibility, cost and value of information of a multicentre randomised controlled trial of intravenous immunoglobulin for sepsis (severe sepsis and septic shock): incorporating a systematic review, meta-analysis and value of information analysis. Health Technol Assess.

[23] Alejandria MM, Lansang MA, Dans LF, Mantaring JB (2013). Intravenous immunoglobulin for treating sepsis, severe sepsis and septic shock. Cochrane Database Syst Rev.

[24] Yang Y, Yu X, Zhang F, Xia Y (2019). Evaluation of the effect of intravenous immunoglobulin dosing on mortality in patients with sepsis: a network meta-analysis. Clin Ther.

[25] Rhodes A, Evans LE, Alhazzani W, Levy MM, Antonelli M, Ferrer R (2017). Surviving sepsis campaign: international guidelines for management of sepsis and septic shock: 2016. Crit Care Med.

[26] National Blood Authority. Criteria for clinical use of immunoglobulin in Australia. Assessed 27 Aug 2021. Available from: https://www.blood.gov.au/ig-criteria.

[27] Heidt S, Roelen DL, Eijsink C, Eikmans M, van Kooten C, Claas FH (2010). Calcineurin inhibitors affect B cell antibody responses indirectly by interfering with T cell help. Clin Exp Immunol.

[28] Heidt S, Roelen DL, Eijsink C, van Kooten C, Claas FH, Mulder A (2008). Effects of immunosuppressive drugs on purified human B cells: evidence supporting the use of MMF and rapamycin. Transplantation.

[29] De Bruyne R, Bogaert D, De Ruyck N, Lambrecht BN, Van Winckel M, Gevaert P (2015). Calcineurin inhibitors dampen humoral immunity by acting directly on naive B cells. Clin Exp Immunol.

